# Supplementary material for: Model-based analysis of experimental data from interconnected, row-configured huts elucidates multifaceted effects of a volatile chemical on Aedes aegypti mosquitoes
Source: Parasit Vectors. 2018 Jun 26;11:365. doi: 10.1186/s13071-018-2919-0 (PMC6019810; doi:10.1186/s13071-018-2919-0)
Supplement: Supplementary file 1 — Table S1. Average Gelman-Rubin statistics across simulated data sets (median and the upper bound of the 95% confidence interval). Figure S1. Correlations between parameter posteriors of model fit on baseline scenario with all parameters estimated. Marginal posteriors are depicted on the diagonals. The numbers on the right of the diagonal depict the Spearman rank correlation coefficients for each side by side comparison. Figure S2. Correlations between parameter posteriors of model fit on baseline scenario with ri fixed (rT: 0.040, r1:0.036, r2: 0.088). Marginal posteriors are depicted on the diagonals. The numbers on the right of the diagonal depict the Spearman rank correlation coefficients for each side by side comparison. Under this parameterization, the movement rate qi is exactly equal to the product xi/ ri. Figure S3. Correlations between parameter posteriors of model fit on low dosage scenario with ri fixed (rT: 0.040, r1:0.036, r2: 0.088). Marginal posteriors are depicted on the diagonals. The numbers on the right of the diagonal depict the Spearman rank correlation coefficients for each side by side comparison. Under this parameterization, the movement rate qi is exactly equal to the product xi/ ri. Figure S4. Correlations between parameter posteriors of model fit on high dosage scenario with ri fixed (rT: 0.040, r1:0.036, r2: 0.088). Marginal posteriors are depicted on the diagonals. The numbers on the right of the diagonal depict the Spearman rank correlation coefficients for each side by side comparison. Under this parameterization, the movement rate qi is exactly equal to the product xi/ ri. Figure S5. Posterior distributions of model parameters fitted to experimental data while fixing the values of ri at the 2.5th percentile of the posterior from the full parameter fit to the baseline data (rT: 0.031, r1:0.027, r2: 0.065). Posteriors are shown for the baseline (gray), low dosage (orange) and high dosage (pink) for the SR-hut (subscript 0) and huts 2 or [file 13071_2018_2919_MOESM1_ESM.docx]

## Additional file 1

**Table S1**. Average Gelman-Rubin statistics across simulated data sets (median and the upper bound of the 95% confidence interval).

|  | **Large sample size scenario** | | **Field scenario** | | |
| --- | --- | --- | --- | --- | --- |
| **Parameter** | **Median** | **Upper bound 95% CI** | | **Median** | **Upper bound 95% CI** |
| *q_2_* | 1.01 | 1.02 | 1.02 | | 1.03 |
| *q_1_* | 1.02 | 1.05 | 1.00 | | 1.01 |
| *q_T_* | 1.01 | 1.03 | 1.00 | | 1.01 |
| *p_1_* | 1.09 | 1.21 | 1.01 | | 1.02 |
| *r_2_* | 1.01 | 1.02 | 1.01 | | 1.02 |
| *r_1_* | 1.07 | 1.18 | 1.01 | | 1.02 |
| *r_T_* | 1.08 | 1.20 | 1.01 | | 1.03 |
| *k_2_* | 1.01 | 1.03 | 1.01 | | 1.01 |
| *k_1_* | 1.08 | 1.20 | 1.01 | | 1.02 |
| *k_T_* | 1.09 | 1.21 | 1.01 | | 1.02 |
| *u* | 1.00 | 1.01 | 1.00 | | 1.01 |


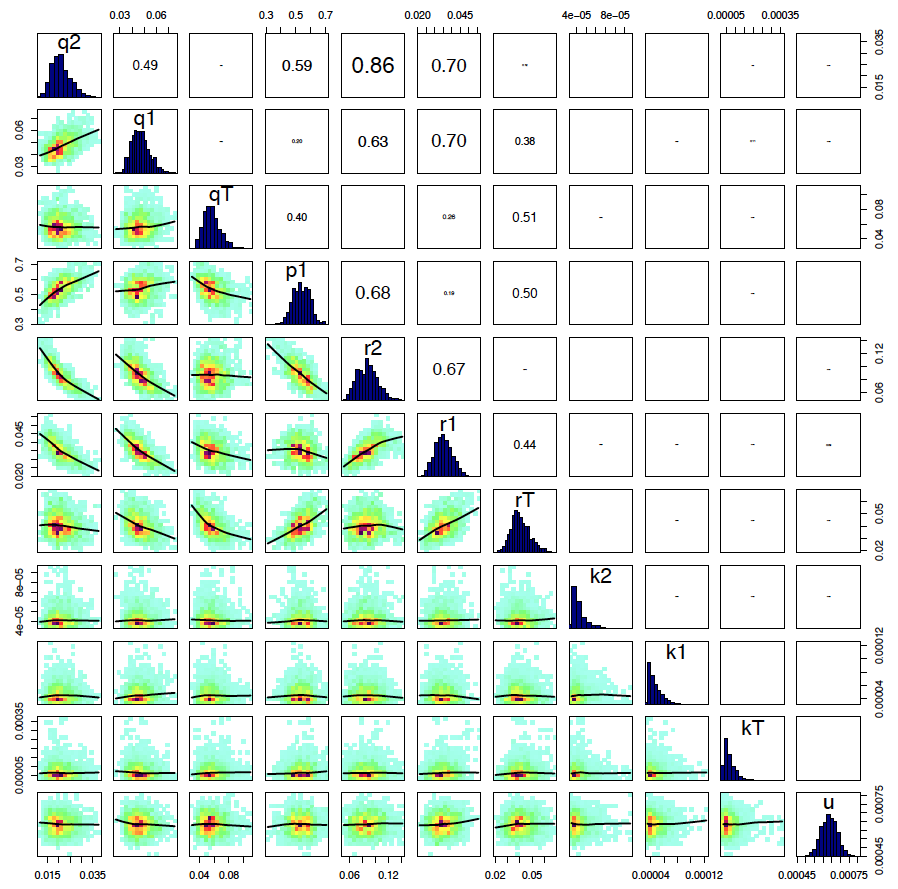


**Figure S1.** Correlations between parameter posteriors of model fit on baseline scenario with all parameters estimated. Marginal posteriors are depicted on the diagonals. The numbers on the right of the diagonal depict the Spearman rank correlation coefficients for each side by side comparison.


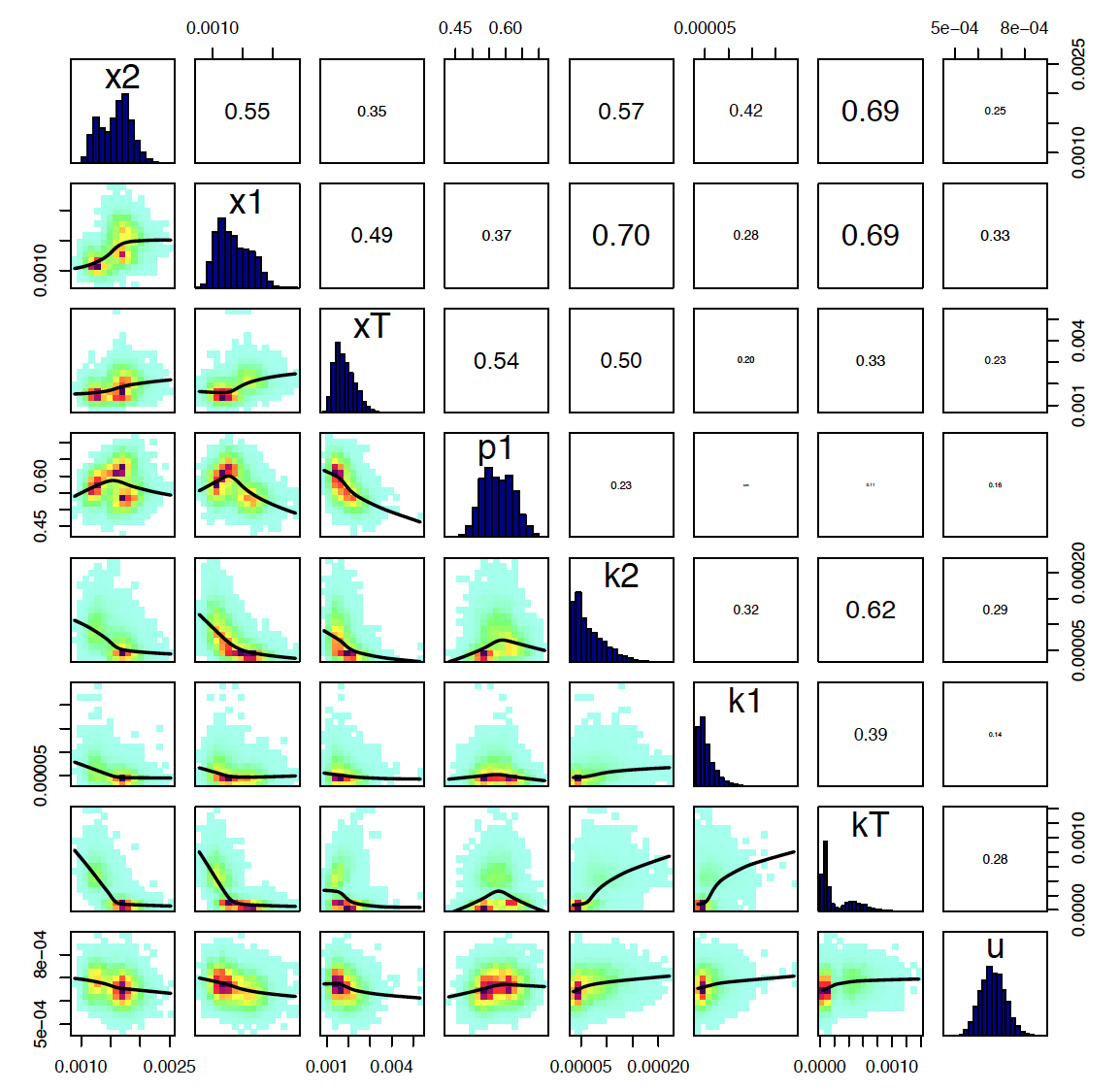


**Figure S2.** Correlations between parameter posteriors of model fit on baseline scenario with *r_i_* fixed (*r*_T_: 0.040, *r*_1_:0.036, *r*_2_: 0.088). Marginal posteriors are depicted on the diagonals. The numbers on the right of the diagonal depict the Spearman rank correlation coefficients for each side by side comparison. Under this parameterization, the movement rate *q_i_* is exactly equal to the product *x_i_ / r_i_*.


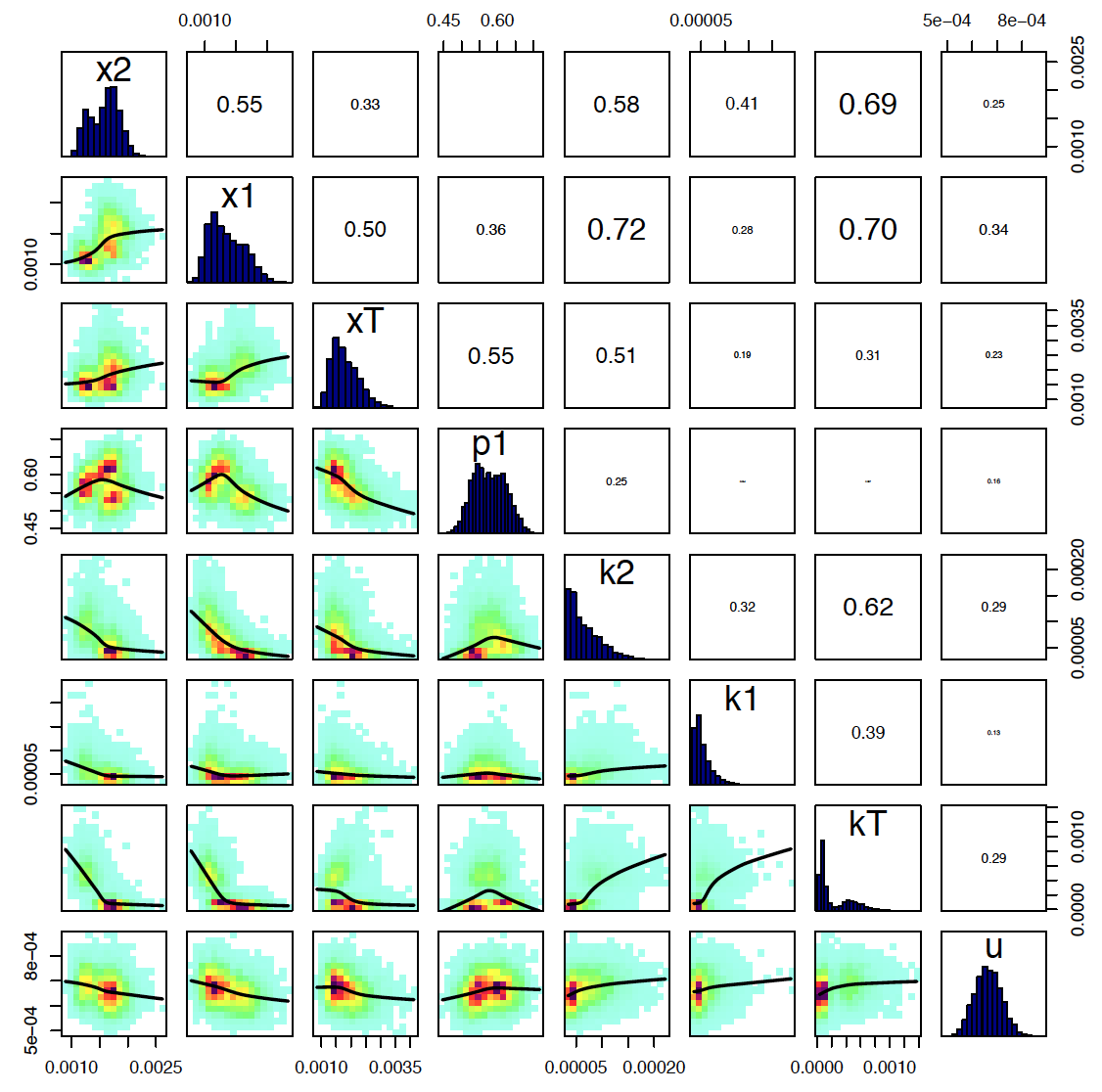


**Figure S3.** Correlations between parameter posteriors of model fit on low dosage scenario with *r_i_* fixed (*r*_T_: 0.040, *r*_1_:0.036, *r*_2_: 0.088). Marginal posteriors are depicted on the diagonals. The numbers on the right of the diagonal depict the Spearman rank correlation coefficients for each side by side comparison. Under this parameterization, the movement rate *q_i_* is exactly equal to the product *x_i_ / r_i_*.


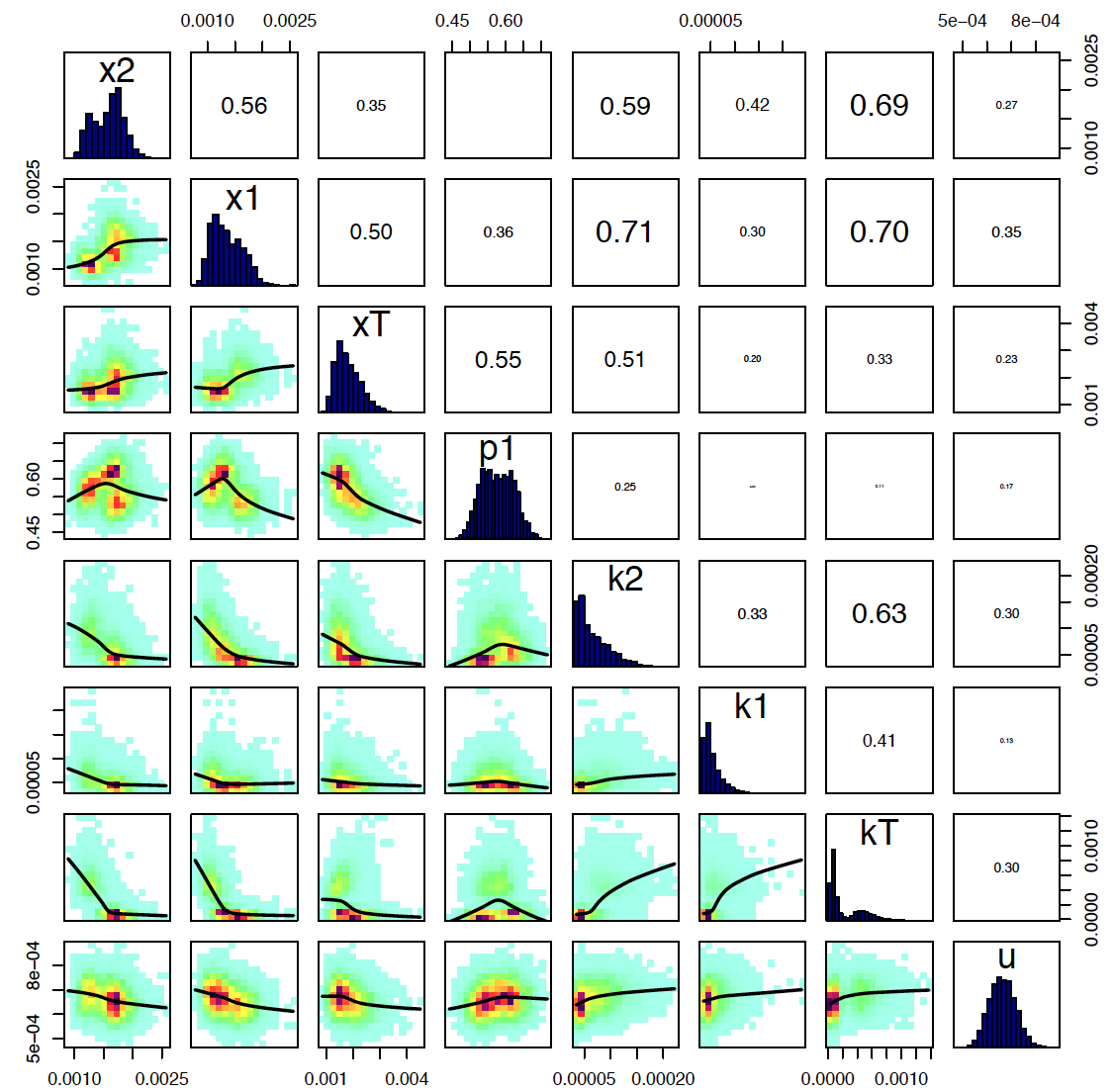


**Figure S4.** Correlations between parameter posteriors of model fit on high dosage scenario with *r*_i_ fixed (*r*_T_: 0.040, *r*_1_:0.036, *r*_2_: 0.088). Marginal posteriors are depicted on the diagonals. The numbers on the right of the diagonal depict the Spearman rank correlation coefficients for each side by side comparison. Under this parameterization, the movement rate *q_i_* is exactly equal to the product *x_i_ / r_i_*.


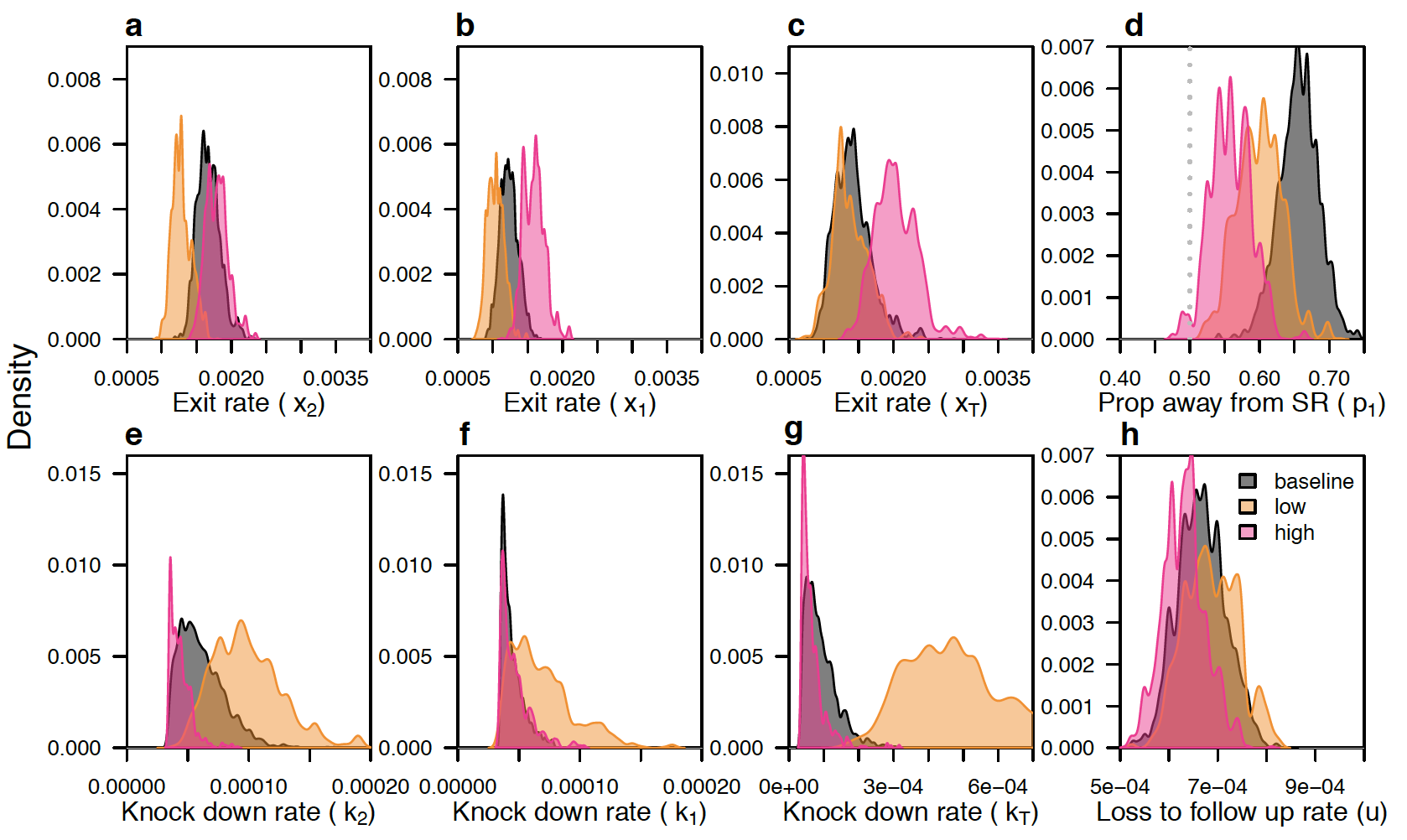


**Figure S5.** Posterior distributions of model parameters fitted to experimental data while fixing the values of *r_i_* at the 2.5th percentile of the posterior from the full parameter fit to the baseline data (*r*_T_: 0.031, *r*_1_:0.027, *r*_2_: 0.065). Posteriors are shown for the baseline (gray), low dosage (orange) and high dosage (pink) for the SR-hut (subscript 0) and huts 2 or 1 removed (subscript 2 and 1, respectively). **a**-**c** rates at which mosquitoes exit the huts, **d** proportion of movement from H_1_ (hut directly adjacent to the treatment hut) away from the SR-product. **e**-**g** knockdown rates and **h** loss to follow-up rates. Under this parameterization, the movement rate *q_i_* is exactly equal to the product *x_i_ / r_i_*.The algorithm was run for 25,000 iterations with a 'burn-in' period of 10,000.


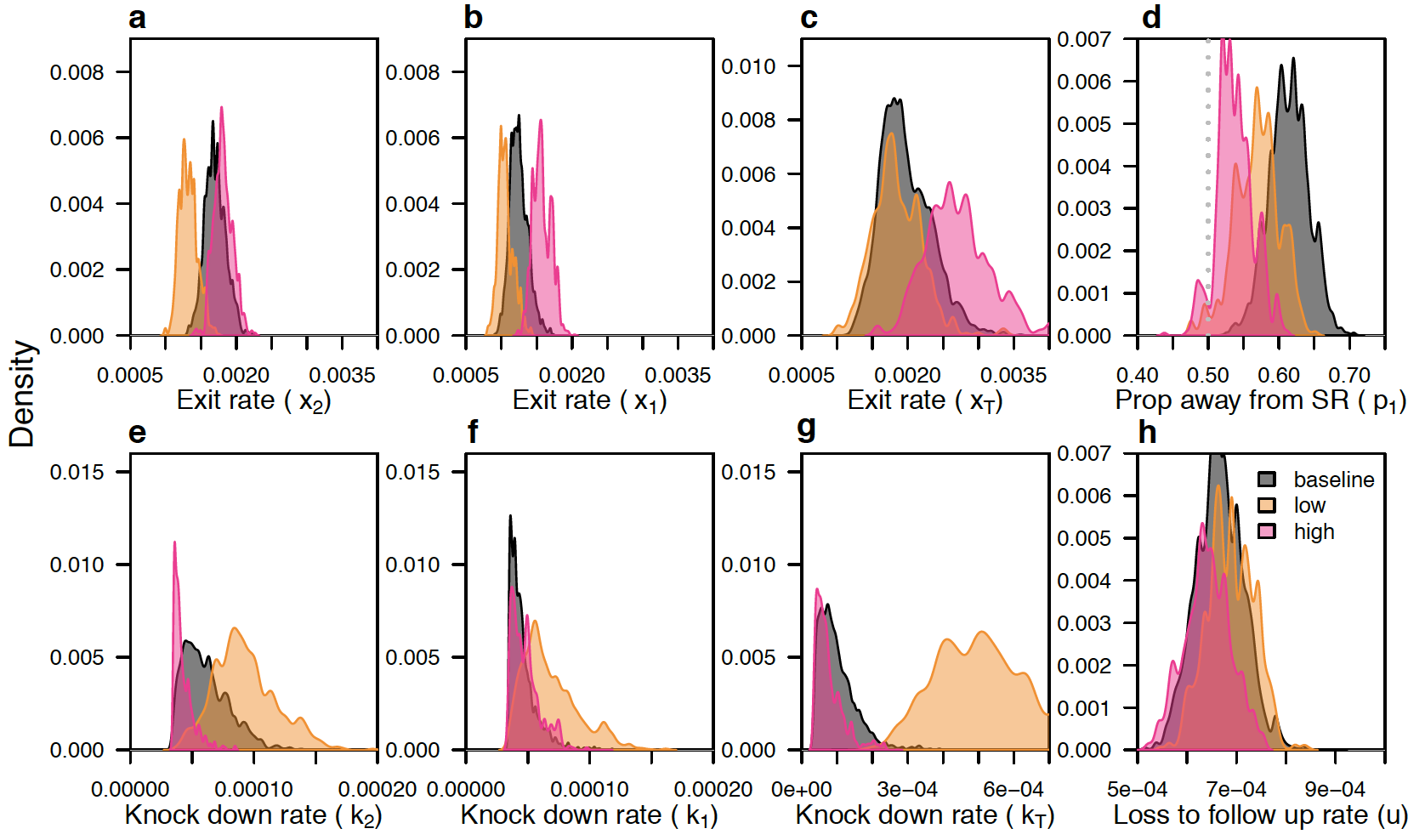


**Figure S6.** Posterior distributions of model parameters fitted to experimental data while fixing the values of *r_i_* at the 97.5th percentile of the posterior from the full parameter fit to the baseline data (*r*_T_: 0.054, *r*_1_:0.040, *r*_2_: 0.11). Posteriors are shown for the baseline (gray), low dosage (orange) and high dosage (pink) for the SR-hut (subscript 0) and huts 2 or 1 removed (subscript 2 and 1, respectively). **a**-**c** rates at which mosquitoes exit the huts, **d** proportion of movement from H_1_ (hut directly adjacent to the treatment hut) away from the SR-product. **e**-**g** knockdown rates and **h** loss to follow-up rates. Under this parameterization, the movement rate *q_i_* is exactly equal to the product *x_i_ / r_i_*. The algorithm was run for 25,000 iterations with a 'burn-in' period of 10,000.


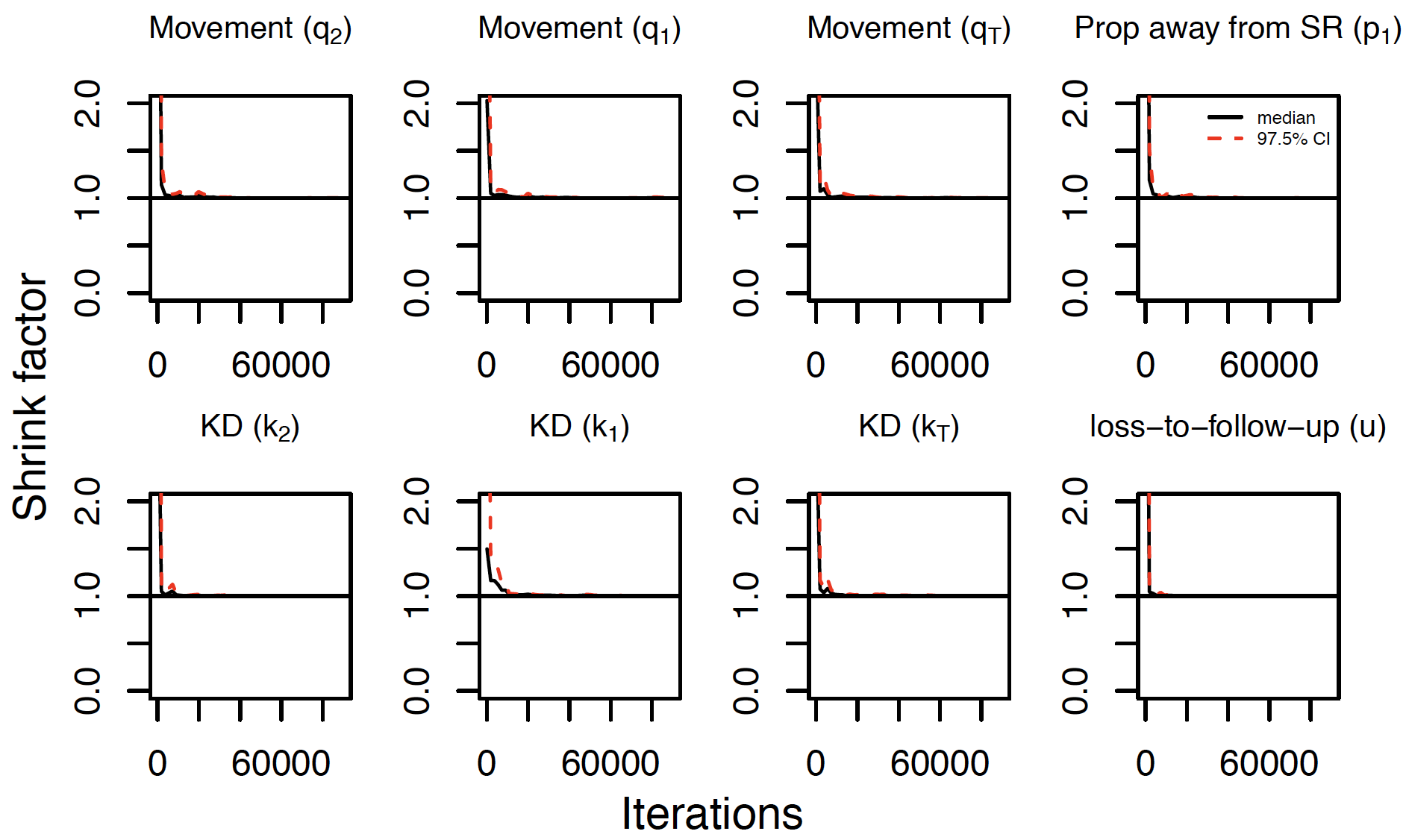


**Figure S7.** Gelman-Rubin convergence diagnostics by iteration for the baseline scenario.


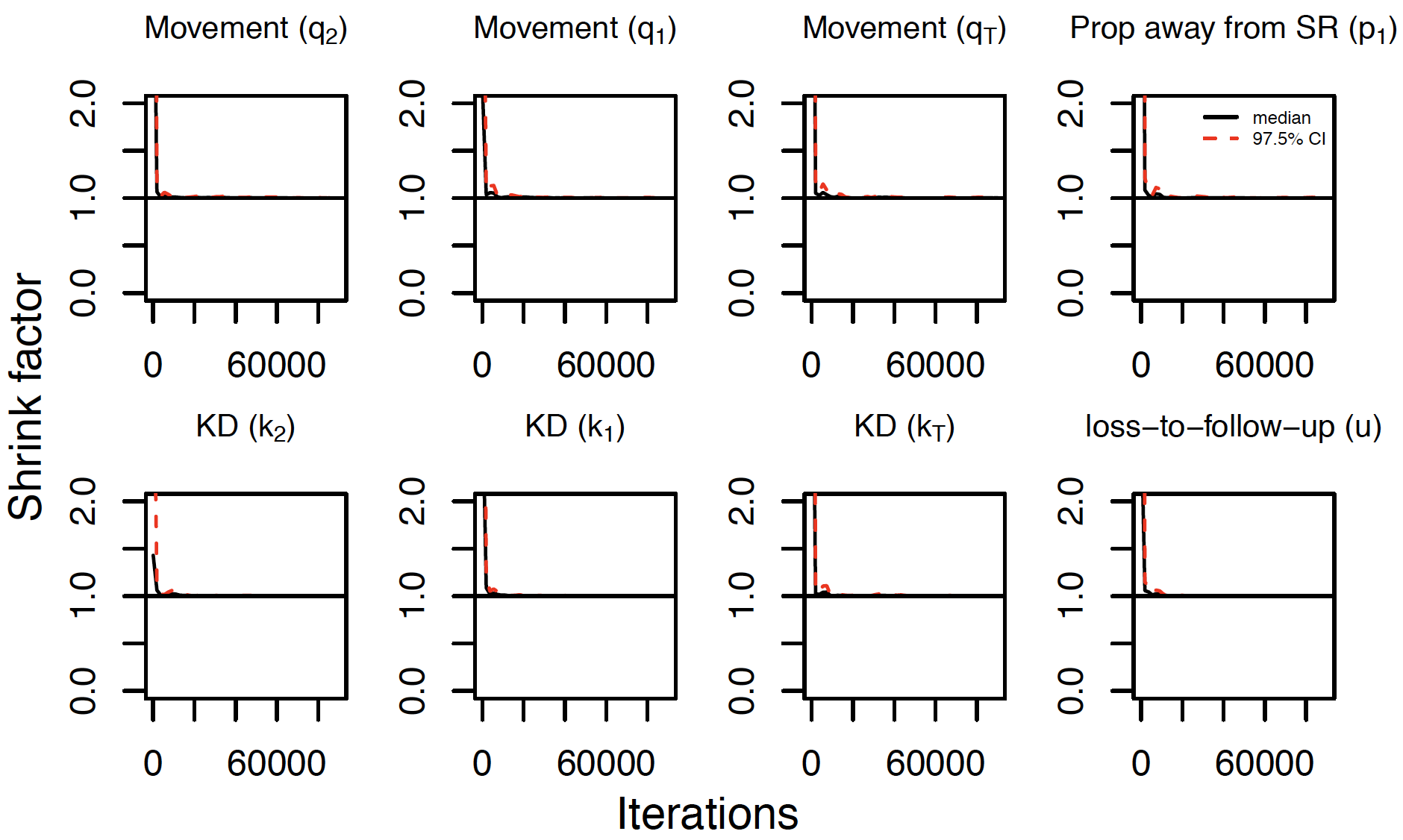


**Figure S8.** Gelman-Rubin convergence diagnostics by iteration for the low dosage scenario.


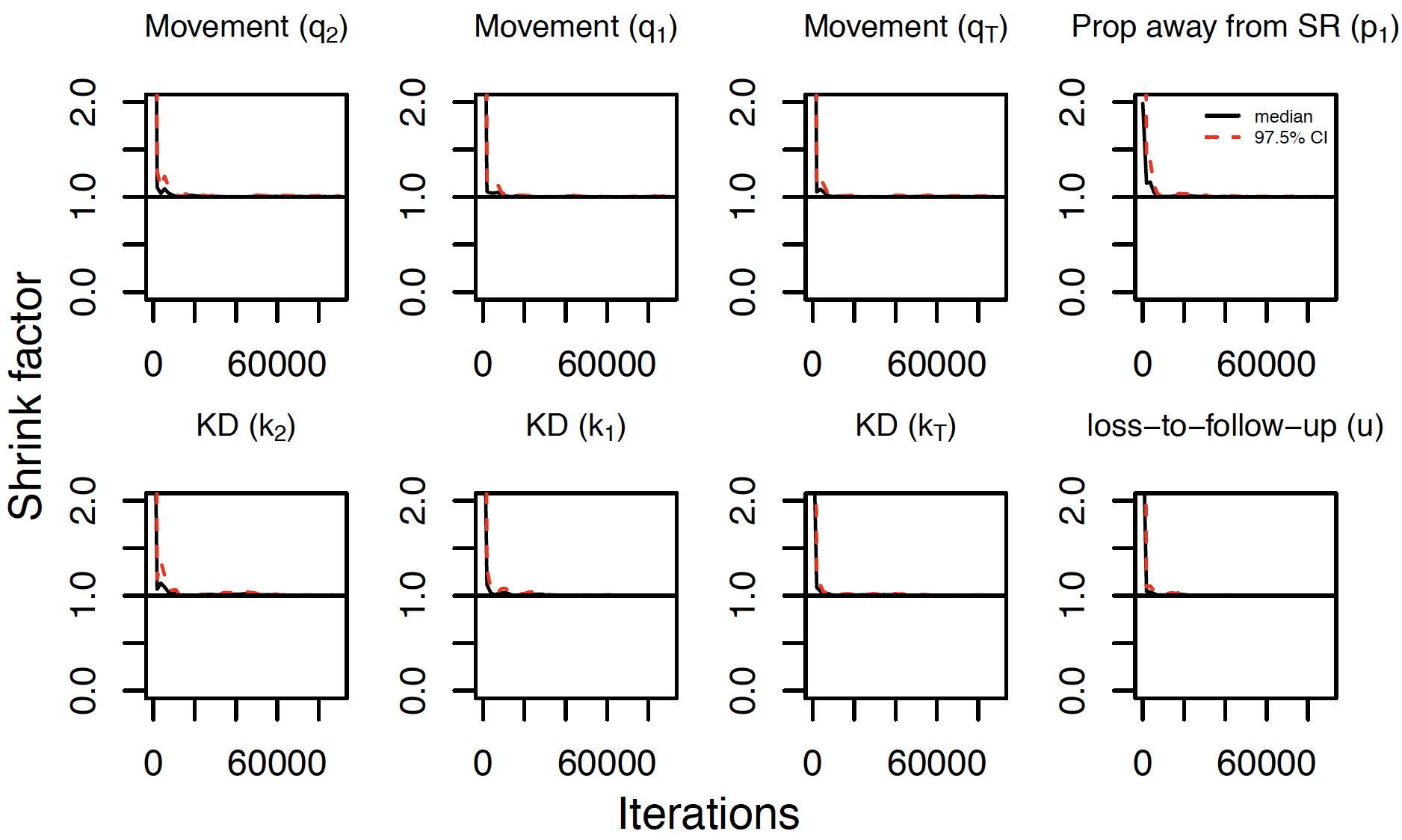


**Figure S9.** Gelman-Rubin convergence diagnostics by iteration for the high dosage scenario.


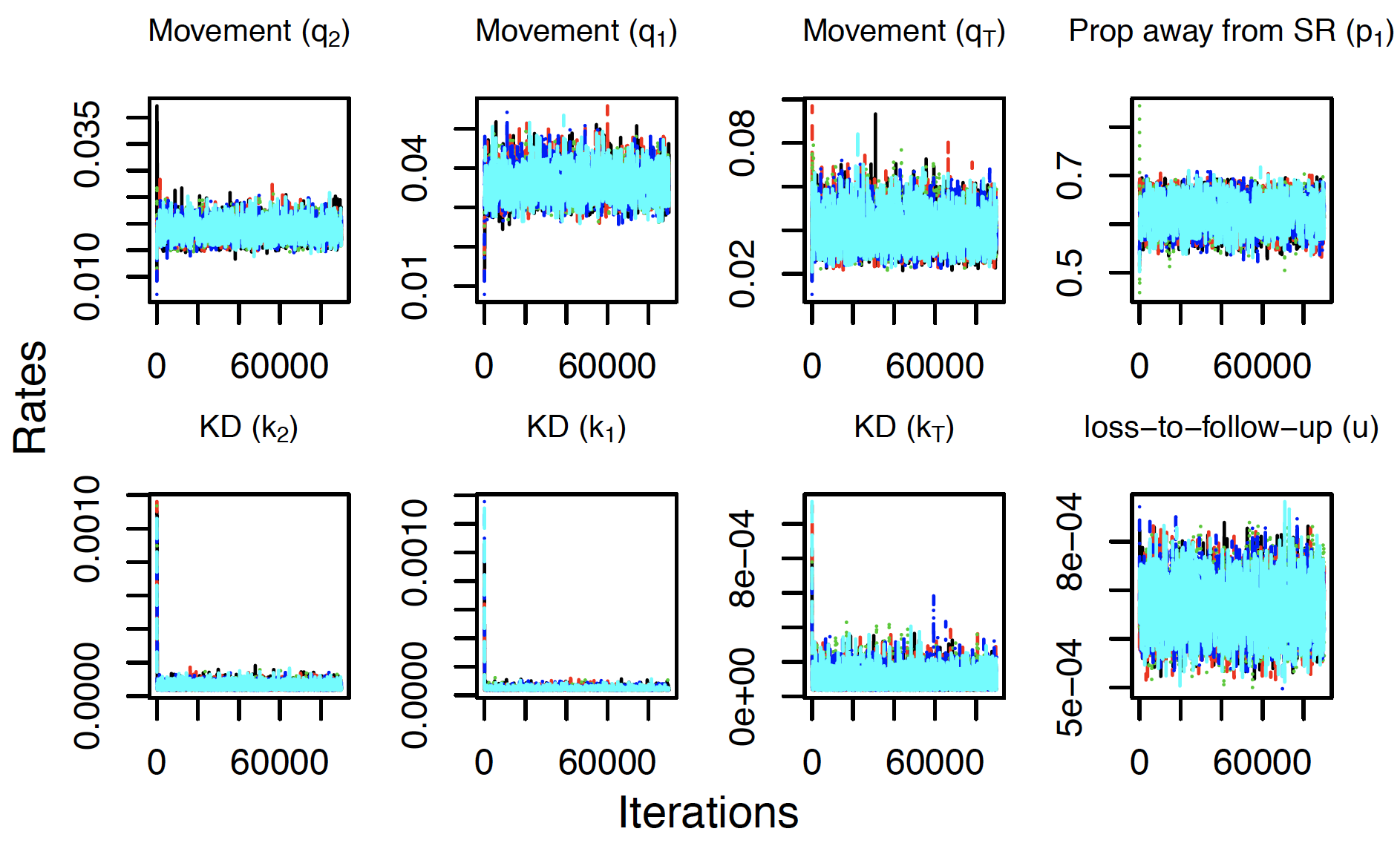
 **Figure S10.** Trace plots for the baseline scenario.


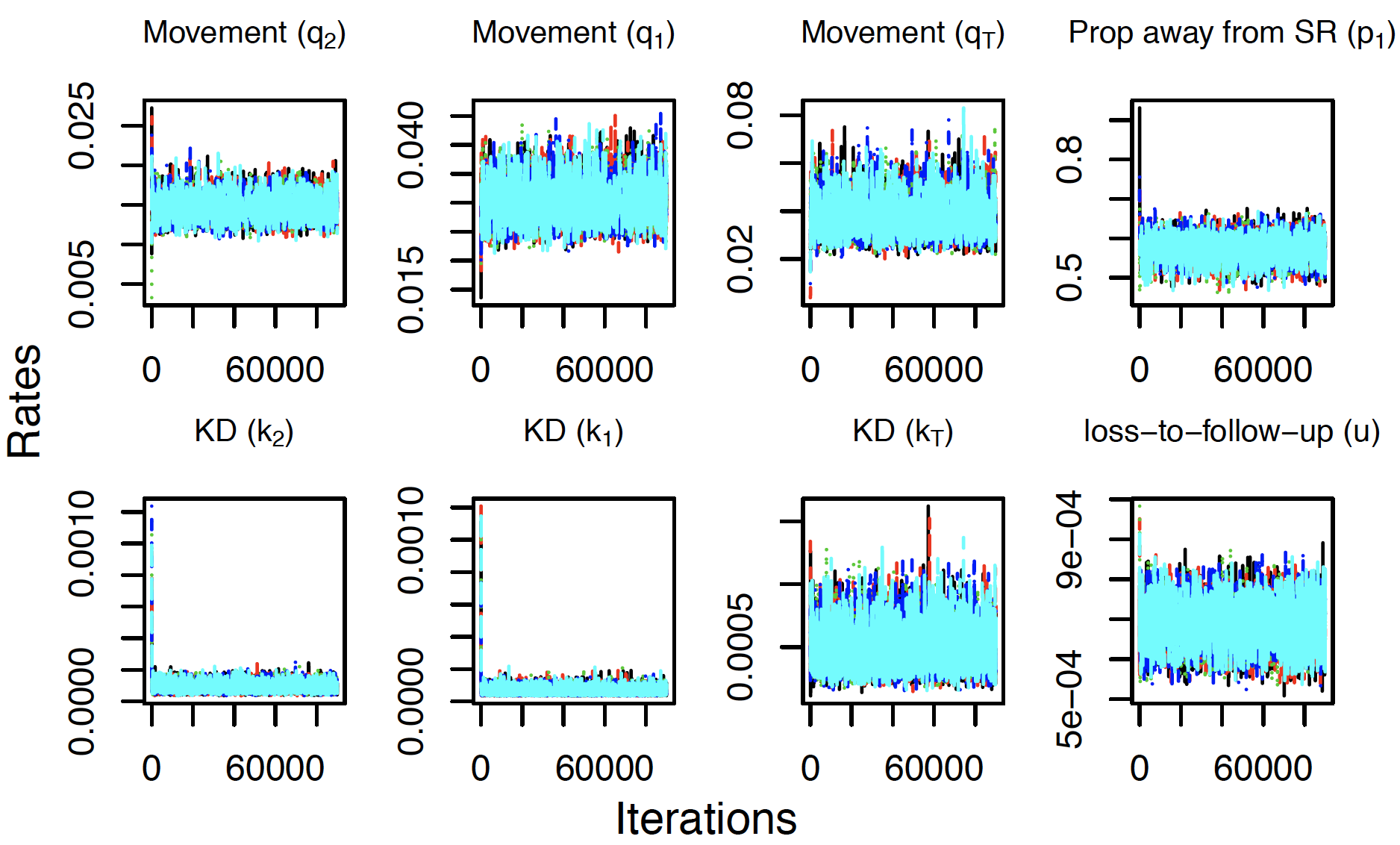


**Figure S11.** Trace plots for the low dosage scenario.


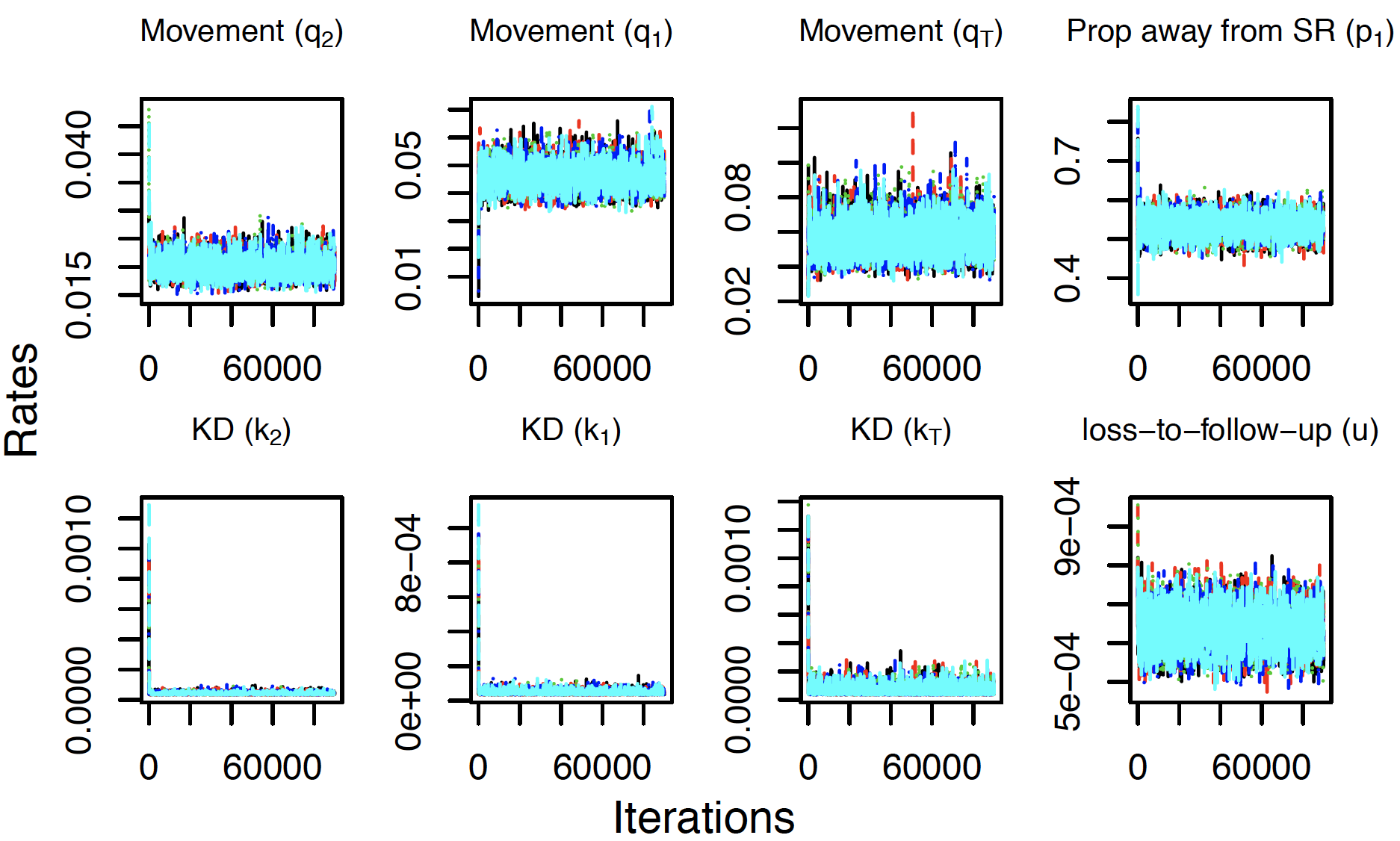


**Figure S12.** Trace plots for the high dosage scenario.
